# Neurally adjusted ventilatory assist (NAVA) allows patient-ventilator synchrony during pediatric noninvasive ventilation: a crossover physiological study

**DOI:** 10.1186/s13054-015-0770-7

**Published:** 2015-02-17

**Authors:** Laurence Ducharme-Crevier, Jennifer Beck, Sandrine Essouri, Philippe Jouvet, Guillaume Emeriaud

**Affiliations:** Pediatric Intensive Care Unit, CHU Sainte-Justine, University of Montreal, 3175 Chemin de la côte Sainte-Catherine, Montreal, QC H3T 1C5 Canada; Keenan Research Centre for Biomedical Science and Li Ka Shing Knowledge Institute of St. Michael’s Hospital, Toronto, Ontario Canada; Pediatric Intensive Care Unit, CHU Kremlin Bicêtre, Université Paris Sud, Le Kremlin Bicêtre, France

## Abstract

**Introduction:**

The need for intubation after a noninvasive ventilation (NIV) failure is frequent in the pediatric intensive care unit (PICU). One reason is patient-ventilator asynchrony during NIV. Neurally adjusted ventilatory assist (NAVA) is a mode of ventilation controlled by the patient’s neural respiratory drive. The aim of this study was to assess the feasibility and tolerance of NIV-NAVA in children and to evaluate its impact on synchrony and respiratory effort.

**Methods:**

This prospective, physiologic, crossover study included 13 patients requiring NIV in the PICU of Sainte-Justine’s Hospital from October 2011 to May 2013. Patients were successively ventilated in conventional NIV as prescribed by the physician in charge (30 minutes), in NIV-NAVA (60 minutes), and again in conventional NIV (30 minutes). Electrical activity of the diaphragm (EAdi) and airway pressure were simultaneously recorded to assess patient-ventilator synchrony.

**Results:**

NIV-NAVA was feasible and well tolerated in all patients. One patient asked to stop the study because of anxiety related to the leak-free facial mask. Inspiratory trigger dys-synchrony and cycling-off dys-synchrony were significantly shorter in NIV-NAVA versus initial and final conventional NIV periods (both *P* <0.05). Wasted efforts were also decreased in NIV-NAVA (all values expressed as median and interquartile values) (0 (0 to 0) versus 12% (4 to 20) and 6% (2 to 22), respectively; *P* <0.01). As a whole, total time spent in asynchrony was reduced to 8% (6 to 10) in NIV-NAVA, versus 27% (19 to 56) and 32% (21 to 38) in conventional NIV before and after NIV-NAVA, respectively (*P* =0.05).

**Conclusion:**

NIV-NAVA is feasible and well tolerated in PICU patients and allows improved patient-ventilator synchronization. Larger controlled studies are warranted to evaluate the clinical impact of these findings.

**Trial registration:**

ClinicalTrials.gov NCT02163382. Registered 9 June 2014.

## Introduction

Respiratory failure is one main reason for admission to the pediatric intensive care unit (PICU) [1]. Mechanical ventilation-related complications occur in 40% of patients [2,3]. Noninvasive ventilation (NIV) aims to minimize those complications while supporting the patient’s breathing [4,5]. However, NIV failure requiring intubation occurs in 19% to 45% of children [6-9] and is associated with a prolonged PICU stay [8-10]. The inability of the ventilator to detect the patient’s breathing efforts crucially limits the efficiency of NIV [11,12]. Patient-ventilator synchronization is critical to reduce the work of breathing [13] and to achieve successful NIV. Obtaining an optimal synchrony during pediatric NIV is a challenge [11]. The presence of leaks at the patient-mask interface, the small tidal volume, and the agitation of the patient impair the detection of the patient breathing effort. Therefore, CPAP (continuous positive airway pressure) is the NIV modality most often used for infants, because asynchronous bilevel pressure ventilation does not seem to offer any advantage [11].

Neurally adjusted ventilatory assist (NAVA) is a mechanical ventilation modality in which the ventilator assists the patient breath in proportion to and in synchrony with the electrical activity of the diaphragm (EAdi) [14]. EAdi is a fast and accurate reflection of phrenic nerve activity arising from central respiratory centers [15]. Six pediatric studies showed improved patient-ventilator interaction with NAVA delivered via endotracheal tube [12,16-20]. As synchronization in NAVA is independent of pressure or flow trigger and independent of leaks [21,22], NAVA has a strong potential to increase NIV efficiency. Few studies have evaluated NAVA in pediatric noninvasive conditions. A preliminary study conducted in six postsurgery children tends to confirm this potential [23].

The main objective of this study is to evaluate the feasibility and tolerance of NIV-NAVA in children treated for respiratory failure of different etiologies in PICU. The secondary objectives are to compare the synchrony and the mean EAdi, as a reflection of patient work of breathing, during conventional NIV and NIV-NAVA. We hypothesized that NIV-NAVA is feasible, improves patient-ventilator synchrony in children compared with conventional NIV, and therefore reduces the patient’s effort.

## Methods

This is a prospective, physiological, crossover study comparing NIV-NAVA versus conventional NIV in children admitted to the PICU for respiratory failure. This study was conducted from October 2011 to May 2013 in the PICU of Sainte-Justine’s Hospital. The protocol was approved by the Ethics Committee of Sainte-Justine Research center (reference 3388). Written informed consent was obtained from the parents or legal tutors. This study follows CONSORT recommendations.

### Study population

Children older than 3 days and younger than 18 years with respiratory failure, expected to require NIV for more than 6 hours, were eligible. Patient screening was done on a daily basis on weekdays by research staff. The exclusion criteria were (a) contraindication to the placement of a new nasogastric tube; (b) hemodynamic instability requiring dopamine ≥5 μg/kg/min, epinephrine, norepinephrine, or dobutamine; (c) severe respiratory instability requiring imminent intubation, according to the attending physician, or FiO_2_ > 60%, or PaCO_2_ > 80 mm Hg on blood gas in the last hour; (d) cardiac patient in the postoperative course; e) all patients without nasogastric tube, as adding a tube for EAdi recording would represent adding an intervention in their care; and (f) absence of parent or tutor for consent. Patients for whom limitation of life-support treatment was discussed were also excluded.

As per these exclusions, we selected a pediatric intensive care population critically ill from a respiratory standpoint, with NIV and nasogastric tube needs for more than 6 hours.

### Study protocol

After enrollment, a specific nasogastric tube equipped with miniaturized electrodes (NAVA catheter, 6, 8, or 12 Fr; Maquet, Solna, Sweden) was installed, by using the “catheter positioning” screen on the Servo I ventilator (v 6.0; Maquet). Patients were initially studied on conventional NIV as per their prestudy settings for 30 minutes, then converted to NIV-NAVA for 60 minutes, and finally returned to conventional NIV for 30 minutes.

### Conventional and NIV-NAVA management

During both conventional NIV periods (before and after NIV-NAVA), the parameters were set as prescribed before the study. The management of NIV, the choice of the ventilator, and that of the airway interface were not guided by written protocol. NIV was always delivered with a ventilator equipped with specific NIV algorithm. With the Servo-I, we usually use the infant mode in children smaller than 10 kg.

For the NAVA period, the patient was switched to a Servo I ventilator. Positive end expiratory pressure (PEEP) and FiO_2_ were not modified. For patient previously ventilated with two levels of pressure, the NAVA level was initially determined to achieve the same peak inspiratory pressure as in the conventional mode. For a patient previously on CPAP, the NAVA level was set to achieve a minimal level of respiratory support (5 cm H_2_O). During the first 30 minutes, the NAVA level could be adjusted based on the respiratory status, to aim for respiratory rate ≤40/minute and ≥12/minute, minimizing respiratory efforts, and to compensate for air leaks when resulting delivered pressure was low (>3 cm H_2_O). The NAVA level was kept constant during the last 30 minutes of the NAVA period. During the entire study, settings modifications by caregivers were permitted and documented.

### Data acquisition and measurements

Baseline patient characteristics, cause and modality of NIV, PIM 2 [24], and PELOD l [25] scores were collected prospectively. SpO_2_, FiO_2_, ventilatory settings, and heart rate were recorded throughout the study period. EAdi and airway pressure (P_vent_) were simultaneously recorded, as previously described [17,26].

### Feasibility and tolerance assessment

Feasibility was assessed by the ability to obtain a correct EAdi signal (compatible with NAVA ventilation), the percentage of time spent in NIV-NAVA mode, and NIV-NAVA interruption. Tolerance was evaluated on the respiratory rate, saturation, FiO_2,_ and EAdi.

### Safety guidelines

To provide safety guidelines, the study protocol had termination criteria including (a) decrease of SpO_2_ < 92% with FiO_2_ > 60% for more than 5 minutes; (b) cardiac rate >180/minute for >10 minutes; (c) respiratory rate >60/minute for more than 10 minutes; (d) uncontrolled agitation; or (e) intolerance suspected by the attending physician.

### Asynchrony analysis

The last 10 minutes of each period were analyzed. The following asynchrony parameters were analyzed: inspiratory trigger dys-synchrony, cycling-off dys-synchrony, and incidence of ineffective efforts and of autotriggered breaths. The total time spent in asynchrony was calculated as the cumulative time spent in each asynchrony form throughout the whole recording time.

The following definitions were used for patient-ventilator interaction [27,28]: (a) inspiratory trigger dys-synchrony: time between the beginning of neural inspiration and the ventilator pressurization; (b) cycling-off dys-synchrony: time lag between the end of neural inspiration activity (70% of peak EAdi) and the end of the inspiratory pressure assistance by the ventilator. The cycling-off dys-synchronies are reported as absolute values as cycling-off dys-synchronies usually have a wide distribution of values with positive or negative values [17,29]; (c) ineffective effort: neural breath not assisted by the ventilator; (d) autotriggered breath: initiation of mechanical assist by the ventilator without an inflection of EAdi. The four types of events were detected breath-by-breath with semiautomatic analysis (one automatic step followed by a manual verification) of EAdi and airway pressure waveforms, comparing the neural timing and the ventilator timing [30]. The inspiratory and cycling-off dys-synchrony can be positive (that is, the ventilator action is delayed compared with EAdi) or negative (that is, the ventilator action is premature in relation to EAdi). The ineffective efforts are expressed in percentage of neural cycles and the autotriggered breaths are expressed in percentage of ventilator breaths. These parameters were measured for all patients during NIV-NAVA period and during conventional NIV periods for patients on the bilevel type of conventional NIV. The total time spent in asynchrony was calculated as the cumulative time spent in each asynchrony form throughout the whole recording time. This global percentage was chosen rather than the Asynchrony Index described by Thille *et al*. [27] because inspiratory and cycling-off dys-synchronies are particularly important in the pediatric population. CPAP periods were not included in synchrony analysis.

To evaluate objectively the ventilatory effort, mean EAdi was calculated as the area under the curve for the entire period of NIV. Two periods (one in initial NIV and one in NIV-NAVA) were excluded from EAdi analysis because of technical problems.

### Statistical analysis

Data are reported as median (interquartile range), unless otherwise specified. Continuous variables were compared between the three periods by using the nonparametric Friedman test and the Wilcoxon Signed-Rank Test for *post hoc* intergroup analysis. Patients in whom CPAP was used during conventional NIV were excluded from the statistical analysis of asynchrony parameters. Level of significance was set to *P* <0.05.

In this convenience sample, inclusion of 15 patients was planned to attempt to represent the PICU population in this feasibility study.

## Results

**Figure 1:**
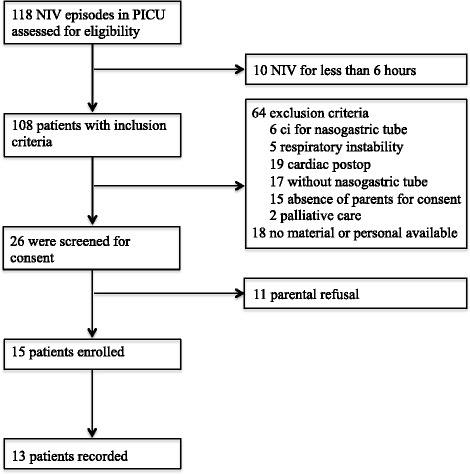
**Patients’ flow charts.**

During the study period, 118 NIV episodes were screened for possible inclusion. Ten patients did not meet the inclusion criteria, 75 patients had exclusion criteria, and for 18 patients, material or personnel for the study was not available (Figure 1). In total, 15 patients were enrolled in the study. Two included patients could not be recorded because of personnel or material unavailability. Thirteen patients were included in the analysis. The patient’s characteristics are summarized in Table 1. The median age was 42 (2 to 109) months. Eight patients were admitted for pneumonia or bronchiolitis. Six patients were taking NIV to facilitate extubation. The ventilator settings are described in Table 2. At baseline, three patients were on CPAP, five on PSV, and five on PCV. Three types of interface were used: four nasopharyngeal tubes, three nasal masks, and six nasobuccal masks. The conventional NIV was delivered with Servo I (*n* = 5), BiPAP version (*n* = 1), VPAP III (*n* = 1), Trilogy (*n* = 3), or babylog 8000 (*n* = 3).

**Table 1:** **Patient characteristics**

**Patient**	**Gender**	**Age (months)**	**Weight (kg)**	**PIM 2 (admission)**	**PELOD**	**After extubation**	**Reason for PICU admission**	**Conventional NIV mode admission**	**Prestudy NIV duration (hours)**
1	M	1.5	4.5	0.3	10	No	Bronchiolitis	CPAP	20
2	F	2.5	2.3	1.5	2	Yes	Bronchiolitis	CPAP	6
3	M	42	15	4.3	1	Yes	Pneumonia	PSV	19
4	M	151	24.7	0.6	3	Yes	Postop scoliosis	PCV	19
5	M	190	55	0.5	3	Yes	Postop scoliosis	PCV	167
6	M	61.5	21.2	0.3	1	No	Pneumonia	PSV	12
7	M	142	20	0.3	3	Yes	Postop scoliosis	PSV	7
8	M	2	4.8	38.5	13	Yes	Heart failure	CPAP	22
9	F	8	6.8	0.2	0	No	Bronchiolitis	PSV	25
10	M	76	14.8	0.3	0	No	Aspiration pneumonia	PCV	13
11	F	46	15.4	0.4	0	No	Pneumonia	PSV	25
12	M	1	3.1	0.6	0	No	Bronchiolitis	PCV	24
13	F	1	3.3	0.31	0	No	Bronchiolitis	PCV	37

PICU, pediatric intensive care unit; NIV, noninvasive ventilation; EAdi, electrical activity of the diaphragm; M, male; F, female; CPAP, continuous positive airway pressure; PSV, pressure support ventilation; PCV, pressure control ventilation.

**Table 2:** **Noninvasive ventilation parameters**

	**Initial conventional NIV period**	**NIV-NAVA period**	**Final conventional NIV period**
**NIV settings**			
NIV mode	3 CPAP, 5 PSV, 5 PCV	13 NAVA	3 CPAP, 5 PSV, 5 PCV
FiO_2_ (%)	35 (27-40)	30 (25-35)	33 (30-36)
Set respiratory rate (n/min)	14 (10-19)	–	14 (10-19)
PEEP (cm H_2_O)	6 (5-7)	6 (5-7)	6 (5-7)
Pressure support (cm H_2_O)	10 (7-12)	–	10 (7-12)
NAVA gain level (cm H_2_O/μV)	–	0.4 (0.3-0.6)	–
**Measured respiratory parameters**			
Respiratory rate (per minute)	41 (31-64)	41 (31-61)	39 (29-63)
Mean airway pressure (cm H_2_O)	12.3 (8.8-14.8)	10.6 (8.1-14.2)	11.9 (9.2-14.1)
Peak EAdi (μV)	13.4 (6.8-35.1)	15.1 (10.0-27.9)	16.1 (7.2-34.4)
End-expiration EAdi (μV)	1.1 (0.3-3.3)	1.2 (0.8-2.8)	1.7 (0.3-3.3)
Mean EAdi (μV)	7.0 (2.2-16.2)	7.6 (6.2-11.4)	7.7 (2.3-16.6)

All values expressed as median and interquartile values. NIV, noninvasive ventilation; CPAP, continuous positive airway pressure; PSV, pressure support ventilation, PCV, pressure control ventilation.

It was possible to obtain an EAdi signal and to provide NIV-NAVA in all patients. No apnea, reversion to backup ventilation, or interruption of NIV-NAVA was observed, despite patient manipulation or sedation. The study was interrupted prematurely for one patient (patient 4). This adolescent was used to chronic NIV with a nasal mask at home during the night, and a change of mask was required for the purpose of the study. During the initial conventional NIV period, he was ventilated by using pressure-control mode, with a PEEP of 6 cm H_2_O, inspiratory pressure of 16 cm H_2_O, respiratory rate of 18 per minute, and FiO_2_ of 21%, resulting in mean EAdi of 3.3 μV, which increased to 7.5 μV during the NAVA period. The patient argued that the new nasobuccal leak-free mask caused him anxiety in both modes, which ultimately led to refusal of any kind of NIV and interruption of the study at the end of the NAVA period. No side effects were reported.

**Figure 2:**
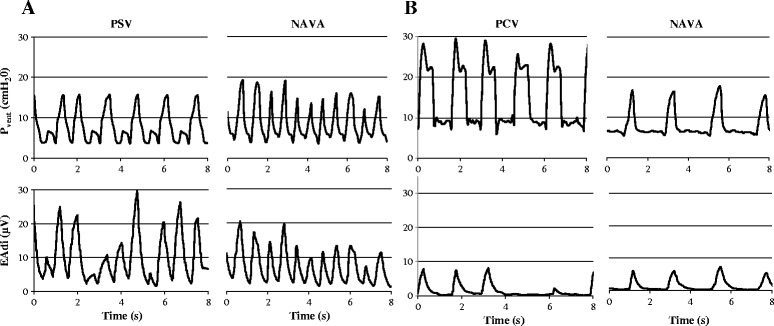
**Example of synchronization between ventilator pressure (P**_**VENT**_ **) and electrical activity of the diaphragm (EAdi) in two children under NIV. (A)** a 1-month-old infant admitted for bronchiolitis on noninvasive Pressure Support Ventilation (PSV) and in NIV-NAVA. **(B)** A 12-year-old postoperative patient with scoliosis on noninvasive Pressure Control (PC) and in NIV-NAVA. We can observe inspiratory and cycling-off asynchrony and autotriggered breaths during conventional NIV.

**Figure 3:**
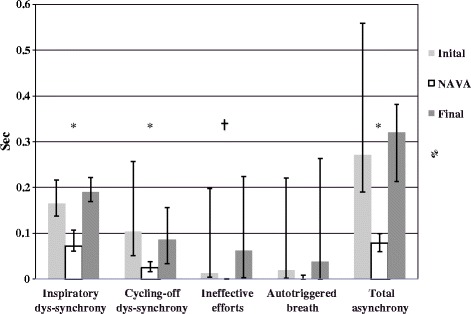
**Inspiratory dys-synchrony (ms), cycling-off dys-synchrony (ms), ineffective efforts (%) and autotriggered breaths (%) in initial conventional NIV, NIV-NAVA, and final conventional NIV.** CPAP periods (*n* = 3) were not included in synchrony evaluation of conventional NIV periods (they could be considered as 100% wasted efforts). *P ≤ 0.05 and ┼*P* < 0.01.

A median of 380 (295 to 617) pneumatic-triggered breaths per period per patient were analyzed. As illustrated in representative patients (Figure 2) and for the entire group (Figure 3), a good synchrony was achieved for all patients in NIV-NAVA, as opposed to conventional NIV. As detailed in Table 3, the total time spent in asynchrony was 8% (6% to 10%) in NAVA, versus 27% (19% to 56%), and 32% (21% to 38%), in conventional NIV before and after periods, respectively (*P* =0.05). Inspiratory trigger dys-synchrony, cycling-off dys-synchrony, and wasted efforts were significantly reduced in NIV-NAVA (*P* <0.05; P <0.05, and P <0.01, Table 3 and Figure 3). The difference for autotriggering did not reach significance.

**Table 3:** **Patient–Ventilator asynchrony parameters in the three NIV conditions**

	**Initial conventional NIV period**	**NIV-NAVA period**	**Final conventional NIV period**	***P*** **value** ^**a**^
Inspiratory trigger synchrony (ms)	164 (137-217)	71 (60-106)	190 (168-222)	0.02^b^
Absolute cycling-off asynchrony (ms)	104 (51-257)	24 (14-38)	86 (33-156)	0.02^b^
Wasted efforts (% neural breath)	12 (4-20)	0 (0-0)	6 (2-22)	<0.01^b^
Autotriggering (% ventilator breath)	2 (0-22)	0 (0-1)	4 (0-26)	0.34
Asynchrony time (% total time)	27 (19-56)	8 (6-10)	32 (21-38)	0.05

All values expressed as median and interquartile values.

^a^Friedman test. The statistical analyses were restricted to the 10 patients in whom conventional NIV was not CPAP.

^b^Significant difference between Initial conventional and NIV-NAVA and between NIV-NAVA and Final Conventional.

**Figure 4:**
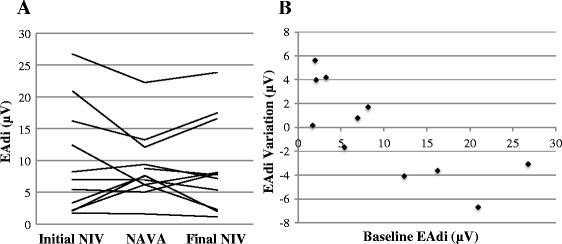
**Comparison of mean EAdi during conventional and NAVA NIV. (A)** Individual mean EAdi during conventional NIV and NIV-NAVA periods. **(B)** The change in mean EAdi observed from conventional NIV to NIV-NAVA period, according to initial EAdi.

The peak and minimal EAdi values were similar in all three periods (*P* =0.3 and *P* =0.9, Table 3). The mean EAdi, a reflection of the patient effort, was similar in all periods. However, the evolution of mean EAdi differed, depending on baseline EAdi, as illustrated in Figure 4. Patients with low baseline mean EAdi (area under the curve below 10 μV, Patients 4, 5, 7, and 13) showed an increase of mean EAdi during NIV-NAVA, whereas patients with higher mean baseline EAdi showed a decrease of EAdi in NAVA.

## Discussion

The current study provides evidence for the feasibility and tolerance of NIV-NAVA in children. Furthermore, NIV-NAVA delivered a well-synchronized support, whereas conventional NIV was associated with important patient-ventilator asynchrony. No difference in patient effort was found among NIV conditions as a whole, even if it tended to decrease in patients with high baseline EAdi.

As dys-synchrony may be associated with negative impact on outcome [27], optimization of patient-ventilator synchrony is probably necessary for NIV success. NIV is increasingly used in PICU [5,31,32]. However, NIV failure is relatively frequent (19% to 45%) [6-9,28,32] and is associated with a longer length of stay than that in patients with invasive ventilation only [5]. To decrease NIV failure, progress of NIV technology is therefore crucial. CPAP currently represents the NIV modality of choice for infants in most PICUs, as bilevel support did not prove any advantage over CPAP [11,33], probably because of the lack of synchronization. Essouri *et al.* [11] described the poor synchrony during conventional NIV in children with upper airway obstruction. Two thirds of breaths were not detected, trigger delays were long (300 ms), and cycling-off was not synchronized [11].

During invasive pediatric ventilation, our team [17] and others [12,19,22] demonstrated the efficacy of NAVA to improve patient-ventilator synchrony. The present study confirms that NAVA also improves patient-ventilator interaction in pediatric NIV conditions. NIV-NAVA did significantly reduce ineffective efforts and dys-synchrony in trigger and cycling-off. The results agree with those of previous adult studies on NIV-NAVA, demonstrating a decrease in premature and delayed cycling, abolishment of ineffective effort, reduction of asynchrony events, and a preserved CO_2_ clearance [34-36]. Our results are also concordant with the only pediatric study of NIV-NAVA, reported by Vignaux *et al.* [23], which documented a significant decrease in ineffective efforts and overall asynchrony index in six postsurgery patients with respiratory distress after extubation. A previous preliminary study by Beck *et al.* [22] showed an adequate synchrony in NIV-NAVA in five premature babies with low birth-weight, despite important leaks.

The present study confirms the benefit of NIV-NAVA on synchrony in a group of critically ill children with different underlying diseases, different ages and weights and different baseline NIV settings, reflecting the actual practice in PICUs.

Inspiratory trigger and cycling-off dys-synchrony are important components of asynchrony in the pediatric population. Children present short inspiratory and expiratory times and delays to activate or inactivate the assistance have a higher proportional significance [17,26]. In our study, these delays contributed markedly to asynchrony during conventional NIV. This asynchrony time was increased by the occurrence of wasted efforts and autocycled breaths. Of note, the total time spent in asynchrony in conventional NIV was similar in younger (≤2 years old) and older children in the present study. In NIV-NAVA, most of these asynchrony events improved, and the total time spent in asynchrony decreased to 8%. Of note, some asynchrony remained in NIV-NAVA, mainly inspiratory trigger delay and cycling-off asynchrony, both of brief duration (71 and 24 ms, respectively). We were not able to record the type of trigger that was activated during NAVA ventilation, so the asynchrony parameters during NAVA include both breaths triggered by the EAdi and the pneumatic triggers. Autotriggered breaths in NAVA may be explained by the pneumatic triggering, which can be activated, besides neural triggering, on a first-come, first-served principle.

Another potential interest of synchrony is the improved airway control. Studies in lambs have shown that conventional NIV is associated with glottal constriction during the inspiratory assist [37]. The authors speculated that the rapid insufflating flow could trigger a reflex constriction of the glottal constrictor muscle. Laryngeal constriction was not observed during NIV-NAVA [38]. In healthy adults, hyperventilation can also result in glottis narrowing and decrease in diaphragmatic activity, whereas normal spontaneous breathing is associated with glottis widening [39]. During NAVA, the inspiratory assistance is in proportion to the patient respiratory drive, and the normal respiratory pattern of the airway and respiratory muscles may be better respected.

Our study reinforces the feasibility of NIV-NAVA in the PICU. In clinical conditions, including the use of sedation, nursing care, physical examination and agitation, there was no interruption related to NAVA technology, and no reversal to backup ventilation. NIV-NAVA is not associated with an increase of invasiveness, as a nasogastric tube is used in most children during NIV. The only premature interruption seemed to be related to anxiety from a leak-free mask, although this was a subjective perception, and we cannot rule out an effect of the NIV algorithm or flow. Any NIV mode has a risk of discomfort [40], and mask failure is close to 15% [41].

The improvement in synchrony may have clinical benefits. Asynchrony has been associated with discomfort, inadequate unloading of work of breathing, and longer ventilatory support [27]. We did not evaluate the impact on discomfort, and the study was not designed to evaluate the NIV success rate. We did not confirm that NIV-NAVA could decrease the mean EAdi, a reflection of the work of breathing. This can be partially explained by the fact that four patients on bilevel pressure ventilation had very low EAdi at baseline, suggesting they may be overassisted during conventional NIV, a condition that has previously been reported in healthy adults, although with different asynchrony patterns [39]. Two of them were in a postoperative state with NIV conducted as a prophylactic measure after extubation. The two other patients (one with heart failure and one with bronchiolitis) were recovering at the time of recording and exhibited very little respiratory efforts. In these conditions, an increase in EAdi during NAVA is expected, because of feedback and absence of overassistance in this mode [42,43]. Contrastingly, all patients with high mean EAdi (>10 μV) exhibited a decrease in EAdi with NAVA. Interestingly, these patients had acute respiratory failure from bronchiolitis (three infants) and pneumonia (one child).

From these observations, we could hypothesize that the mode of ventilation NAVA may allow the patient to regulate his or her work of breathing. This also suggests that future trials that will evaluate the clinical benefits of NAVA should focus mostly on patients in whom a clear benefit is expected; this may include particularly patients with significant respiratory failure (or high EAdi). It is important to note that the cutoff value of 10 μV is referring not to peak EAdi but to the mean EAdi. The mean EAdi (the area under the curve for the entire recording) was chosen to represent all components of diaphragm activity (inspiration, expiration, frequency) for the entire period (and not limited to a selected period). Inspiratory peak EAdi values in critically ill children have recently been described by our team [17,18,44], and in the recovery phase, median (interquartile) observed peak EAdi values were 13 (8 to 21) μV [44], whereas the area under the curve for EAdi was 6 (2 to 9) μV (unpublished data).

Besides patient’s condition, the setting of NAVA level also influences EAdi. The NAVA level was adjusted based on the respiratory status, as in our clinical practice, and a different NAVA level could have resulted in other EAdi profiles. In a feasibility study in rabbits, the NAVA level had to be increased to achieve a similar unloading, as compared with the pre-extubation condition, mainly because of major air leaks [21].

Several limitations of the study must be outlined. This feasibility study included a limited number of patients in a single PICU, and a majority of NIV-exposed patients during the period were excluded, thereby limiting ability to generalize our findings. The population was intentionally heterogeneous, and representative of various ages and pathologies. The sample size does not permit to assess differences among these groups, but the study results provide further evidence regarding NAVA feasibility, which will permit us to prepare and justify for future studies in these groups. The study included some patients with significant distress, but also patients with prophylactic NIV, in whom few signs of respiratory failure were present. This may have diminished the power to detect a decrease in the work of breathing. We included patients on CPAP, even though no synchrony can be analyzed, because CPAP is the primary NIV option for infants in our PICU. We therefore did not want to exclude those patients from this evaluative study. The study duration was limited to a 2-hour period, to limit period effect. Future longer-term evaluation is therefore necessary. We did not evaluate the impact of the NIV mode on the delivered pharyngeal pressure or tidal volumes, but the EAdi evolution provided some indications on the work of breathing changes.

## Conclusion

NIV-NAVA is feasible and well tolerated in critically ill children. It improves patient-ventilator synchrony when compared with conventional NIV. Further studies are warranted to evaluate the clinical impacts of NIV-NAVA, especially on NIV success, ventilator-support duration, and comfort.

## Key messages

- Asynchrony occurs during almost a third of the time during pediatric conventional noninvasive ventilation.
- Noninvasive NAVA allows us to support the ventilation in synchrony with patient efforts.
- The potential of NAVA to improve noninvasive ventilation success must be studied in children.

## Abbreviations

CPAP: continuous positive airway pressure
EAdi: electrical activity of the diaphragm
F: female
M: male
NAVA: neurally adjusted ventilatory assist
NIV: noninvasive ventilation
PC: pressure control
PEEP: positive end-expiratory pressure
PELOD: pediatric logistic organ dysfunction
PICU: pediatric intensive care unit
PIM 2: pediatric Index of Mortality 2
PSV: pressure support ventilation
Pvent: airway pressure
